# Frequent horizontal and mother-to-child transmission may contribute to high prevalence of STLV-1 infection in Japanese macaques

**DOI:** 10.1186/s12977-020-00525-1

**Published:** 2020-06-23

**Authors:** Megumi Murata, Jun-ichirou Yasunaga, Ayaka Washizaki, Yohei Seki, Madoka Kuramitsu, Wei Keat Tan, Anna Hu, Kazu Okuma, Isao Hamaguchi, Takuo Mizukami, Masao Matsuoka, Hirofumi Akari

**Affiliations:** 1 Center for Human Evolution Modeling Research, Primate Research Institute, Kyoto University, 41-2 Kanrin, Inuyama, 484-8506 Japan; 2 Laboratory of Virus Control, Institute for Frontier Life and Medical Sciences, Kyoto University, 53 Shogoin-Kawahara-cho, Sakyo-ku, Kyoto, 606-8507 Japan; 3 Department of Hematology, Rheumatology and Infectious Disease, Graduate School of Medical Sciences, Kumamoto University, 1-1-1 Honjo Chuo-ku, Kumamoto, 860-8556 Japan; 4 Department of Safety Research on Blood and Biological Products, National Institute of Infectious Diseases, 4-7-1 Gakuen, Musashimurayama, Tokyo, 208-0011 Japan; 5 Laboratory of Infectious Disease Model, Institute for Frontier Life and Medical Sciences, Kyoto University, 53 Shogoin-Kawahara-cho, Sakyo-ku, Kyoto, 606-8507 Japan

**Keywords:** STLV-1, Japanese macaques, Prevalence, Antibody titer, Proviral load, Mother-to-child transmission, Horizontal transmission

## Abstract

**Background:**

Simian T-cell leukemia virus type 1 (STLV-1) is disseminated among various non-human primate species and is closely related to human T-cell leukemia virus type 1 (HTLV-1), the causative agent of adult T-cell leukemia and HTLV-1-associated myelopathy/tropical spastic paraparesis. Notably, the prevalence of STLV-1 infection in Japanese macaques (JMs) is estimated to be > 60%, much greater than that in other non-human primates; however, the mechanism and mode of STLV-1 transmission remain unknown. The aim of this study is to examine the epidemiological background by which STLV-1 infection is highly prevalent in JMs.

**Results:**

The prevalence of STLV-1 in the JMs rearing in our free-range facility reached up to 64% (180/280 JMs) with variation from 55 to 77% among five independent troops. Anti-STLV-1 antibody titers (ABTs) and STLV-1 proviral loads (PVLs) were normally distributed with mean values of 4076 and 0.62%, respectively, which were mostly comparable to those of HTLV-1-infected humans. Our initial hypothesis that some of the macaques might contribute to frequent horizontal STLV-1 transmission as viral super-spreaders was unlikely because of the absence of the macaques exhibiting abnormally high PVLs but poor ABTs. Rather, ABTs and PVLs were statistically correlated (p < 0.0001), indicating that the increasing PVLs led to the greater humoral immune response. Further analyses demonstrated that the STLV-1 prevalence as determined by detection of the proviral DNA was dramatically increased with age; 11%, 31%, and 58% at 0, 1, and 2 years of age, respectively, which was generally consistent with the result of seroprevalence and suggested the frequent incidence of mother-to-child transmission. Moreover, our longitudinal follow-up study indicated that 24 of 28 seronegative JMs during the periods from 2011 to 2012 converted to seropositive (86%) 4 years later; among them, the seroconversion rates of sexually matured (4 years of age and older) macaques and immature macaques (3 years of age and younger) at the beginning of study were comparably high (80% and 89%, respectively), suggesting the frequent incidence of horizontal transmission.

**Conclusions:**

Together with the fact that almost all of the full-adult JMs older than 9 years old were infected with STLV-1, our results of this study demonstrated for the first time that frequent horizontal and mother-to-child transmission may contribute to high prevalence of STLV-1 infection in JMs.

## Background

Primate T-cell leukemia virus is classified into the Deltaretrovirus genus, which includes simian T-cell leukemia virus (STLV) and human T-cell leukemia virus (HTLV). The first human retrovirus, HTLV-1, was identified in 1980 [1, 2], even though the disease entity of adult T-cell leukemia (ATL) had been described in Japan before the identification of this virus [3]. Eventually, HTLV-1 was found to be the causative agent of not only ATL but also HTLV-1-associated myelopathy (HAM)/tropical spastic paraparesis (TSP) [2, 4–6]. It is estimated that 10–20 million people worldwide are infected with HTLV-1 [7]. HTLV-1 is endemic in southern Japan, the Caribbean, Central and South America, and intertropical Africa [7, 8]. An estimated one million people in Japan are thought to be HTLV-1 carriers, corresponding to 1% of the total population [7, 9, 10]. In most cases, HTLV-1 infection remains asymptomatic, whereas 5% of the carriers develop ATL or HAM/TSP [11–18].

As a counterpart of HTLV, STLV is prevalent among a variety of non-human primates in Asia and Africa but not in America [19–23]. STLV-1 and STLV-2 are closely related with HTLV-1 and HTLV-2 [24–26]. A third subspecies, STLV-3, was isolated from an Eritrean sacred baboon (*Papio hamadryas*) and a red-capped mangabey (*Cercocebus torquatus*) [27, 28]. A recent report showed that STLV-4 was isolated from gorillas and that the virus was endemic to gorillas [29]. It has been reported that STLVs originated from African non-human primates are pathogenic to the natural hosts and are associated with leukemia/lymphoma [30–33]. The zoonotic STLV transmission to humans is likely caused by hunting and severe bites of non-human primates [20, 34–38].

Japanese macaques (JMs: *Macaca fuscata*) inhabit widespread of Japan except Hokkaido and Okinawa. Watanabe et al. reported that the sequence homology of JM STLV-1 genome to that of HTLV-1 was 90% [24]. Given this genetic similarity, it had been suspected that zoonotic STLV-1 transmission might be, at least in part, the cause of HTLV-1 dissemination among Japanese. However, subsequent analysis between HTLV-1 isolated from Japanese and STLV-1 from JMs demonstrated that the STLV-1 was phylogenetically distinct from the HTLV-1 [39]. Furthermore, no geographical deviation in terms of the distribution of STLV-1-infected JMs is observed, which is in contrast with the case of HTLV-1-infected Japanese [40, 41]. From these findings, it is concluded that the HTLV-1 is originated in Mongoloid people moving from North Asia but not from the JM STLV-1 [39, 42].

Notably, a high proportion (60% on average) of JMs is reportedly infected with STLV-1, whereas the prevalence of STLV-1 in other natural hosts among non-human primates, including Asian macaques, is generally much lower [19, 41, 43–53]. The reason for the abnormally high prevalence still remains unknown. In this study we sought to examine the epidemiological background as well as mechanism and mode of the STLV-1 transmission by which STLV-1 is highly prevalent among JMs.

## Methods

### Animals

JMs bred and reared in the free-range facility of the Primate Research Institute, Kyoto University (KUPRI) were used in this study. All the troops were isolated and had no physical connection with each other. All animal experiments were approved by the Animal Welfare and Animal Care Committee of KUPRI (approval numbers: 2014–092, 2015-040, and 2016-135) and were conducted in accordance with the Guidelines for Care and Use of Nonhuman Primates (Version 3) by the Animal Welfare and Animal Care Committee of KUPRI.

### Preparation of plasma and peripheral blood mononuclear cells (PBMCs)

Blood samples were collected from JMs at the routine health checkups carried out during the periods from 2011 to 2012 and from 2015 to 2016 under ketamine anesthesia with medetomidine, followed by administration of its antagonist, atipamezole, at the end of the procedure. PBMCs were separated from blood samples with Ficoll-paque PLUS (GE Healthcare, Buckinghamshire, UK) by density gradient centrifugation. Plasma and PBMCs were frozen at − 80 °C until use.

### Titration of the STLV-1-specific antibody

Plasma samples were evaluated for STLV-1-specific antibody titers (ABTs) with a particle agglutination (PA) assay using Serodia-HTLV-1 (Fujirebio Inc. Tokyo, Japan) as previously described [52]. The plasma cut-off titer was a 1:16 dilution. In case of the macaques whose blood samples were positive for the antibody while negative for the proviral DNA were retested for the PA assay with and without absorption procedure according to the manufacturer’s instructions. Only the individuals whose antibody positivity was confirmed by the absorption procedure were indicated as positive for the antibody while negative for the proviral DNA.

### Quantification of STLV-1 proviral DNA loads (PVLs)

Cellular DNA was purified from PBMCs via a QIAamp DNA Blood Mini Kit (QIAGEN, Hilden, Germany), according to the manufacturer’s instructions. The DNA samples were employed for the measurement of STLV-1 PVLs via a real-time PCR of the STLV-1 tax and RAG1 genes of JMs as previously described [52]. PCR was performed using Thunderbird Probe qPCR mix (Toyobo, Osaka, Japan). The following primers and probes were used: for RAG1, RAG1-2F (CCCACCTTGGGACTCAGTTCT), RAG1-2R (CACCCGGAACAGCTTAAATTTC), and a RAG1 probe (5′-FAM CCCCAGATGAAATTCAGCACCCATATA TAMRA-3′); for tax, STLV-1 tax-F2 (CTACCCTATTCCAGCCCACTAG), STLV-1 tax-R3 (CGTGCCATCGGTAAATGTCC), and an STLV-1 tax probe (5′-FAM CACCCGCCACGCTGACAGCCTGGCAA TAMRA-3′), respectively. Copy number of STLV-1 proviral DNA per cell was standardized with that of the RAG1 gene. The detection limit of PVLs was 0.01%.

### Sequence analysis of STLV-1 3′ LTR, tax and env regions

The 3′ LTR, tax and env regions of the viral genome were amplified by using KOD-FX neo polymerase kit (Toyobo, Osaka, Japan) according to the manufacturer’s protocol. The thermal cycle condition for 3′ LTR and env region was as follows; for 1st PCR, 94 °C for 2 min followed by the 5 cycles of 98 °C for 10 s and 68 °C for 5 min, and the 23 cycles of 98 °C for 15 s, 60 °C for 15 s, and 68 °C for 5 min. For 2nd PCR, 94 °C for 2 min followed by the 45 cycles of 98 °C for 15 s, 60 °C for 15 s, and 68 °C for 4 min. For tax region, 94 °C for 2 min followed by the 50 cycles of 98 °C for 15 s, 60 °C for 15 s, and 68 °C for 2 min.

PCR products were purified using QIAquick 96 PCR purification kit (QIAGEN, Hilden, Germany). The amplified fragments were directly sequenced by using BigDye ver 3.1 cycle sequencing kit (Applied Biosystems, Foster City, USA) and an Applied Biosystems 3730 DNA analyzer. Alignment of the determined sequences was analyzed by genetyx software. The following primers were used; for 3′LTR region; 7487F (CCCAGAGAACCTCTAAGACCCT), 8937R (TCAGACGTGAATGAAAGGGAAAG), and sequencing; 8152F (CAAGGCCTACCATCCCTCTT), and for tax region, 1st PCR primers; 5021F (GGAAAGGACCACAGGARGC), 8937R (TCAGACGTGAATGAAAGGGAAAG), 2nd PCR primers; 5524F (CTTAGGATGCCAATCATGGA), 8619R (CCTGTTGTTTTATTGAGCTGTATGC), and sequencing; 7487F (CCCAGAGAACCTCTAAGACCCT) and 7742R (TACATGCAGACAACGGAGTTTCC), and for env region, 1st PCR primers; 3158F2 (CCCAGGACAAAACTCARCAA), 7412R2 (CCGARCATAGTCCCCCAGAGA), 2nd PCR primers; 4173F3 (CCTGCCCCGCCTACTATCRC), 6672R (AAGCAATGTGGTCGCAGTAAC), and sequencing; 5012F (GGAAAGGACCACAGGARGC), 5524F (CTTAGGATGCCAATCATGGA), 5975F (CCCCACCTGACGYTACCATT), respectively.

### Statistical analyses

We tested the normal distribution of the data and applied parametric or non-parametric methods according to the experiment. Pearson’s correlation coefficient was employed for correlation of two parameters, and two-tailed Student’s t-tests were employed for comparison of two groups. For multiple comparisons with more than two groups, a one-way ANOVA with Tukey’s multiple comparison test was used.

## Results

To validate the STLV-1 prevalence in JMs, we first examined for positivity of the anti-STLV-1 antibody in plasma of 280 JMs from five independent troops originating from inhabitants of different areas in Japan. The plasma samples were obtained from JMs at the routine health checkups during the period from 2015 to 2016. We found that 180 of 280 macaques (64%) were seropositive for STLV-1 (Table 1), which was generally consistent with previous reports [41, 43, 44, 52]. We then determined the variation in the seroprevalence among the troops. The numbers of seropositive individuals were 59, 17, 36, 34, and 34, with a frequency of 68%, 55%, 63%, 56%, and 77%, respectively, for each troop (Table 1). In addition, the rearing population density in the free-range facility differed in each troop but was not correlated with the seroprevalence, suggesting that the density did not influence the high STLV-1 prevalence (Table 1).

**Table 1:** Seroprevalence in JMs and other parameters having the possibilities of affecting seroprevalence in each troop

	Troop A	Troop B	Troop C	Troop D	Troop E	Total
Number of individuals	87	31	57	61	44	280
STLV-1 seroprevalence						
(Number of infected/uninfected individuals)	68% (59/28)	55% (17/14)	63% (36/21)	56% (34/27)	77% (34/10)	64% (180/100)
Age in average	5.7	4.5	4.2	6.5	5.0	5.5
Rearing area (m^2^)	8500	3400	850	730	1200	14,680
Area per individuals (m^2^)	97.7	109.7	14.9	12.2	27.3	52.6

**Fig. 1:**
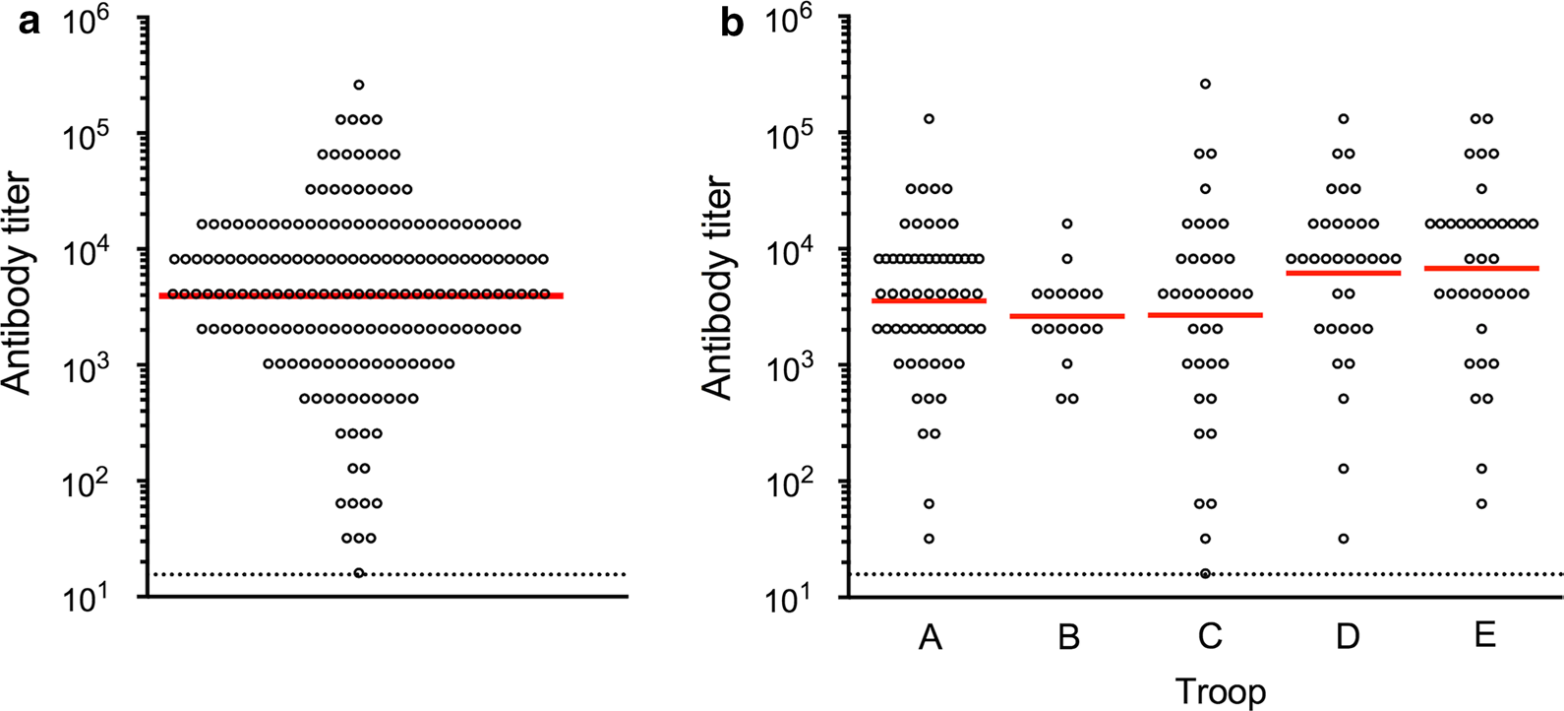
Distribution of anti-STLV-1 antibody titers (ABTs) in seropositive JMs. Distribution of ABTs in all seropositive cohorts JMs (**a**) and in each troop (**b**) is indicated. The dotted line shows the detection limit of the ABT (16), and the red line indicates the geometric mean of the ABT distribution

**Fig. 2:**
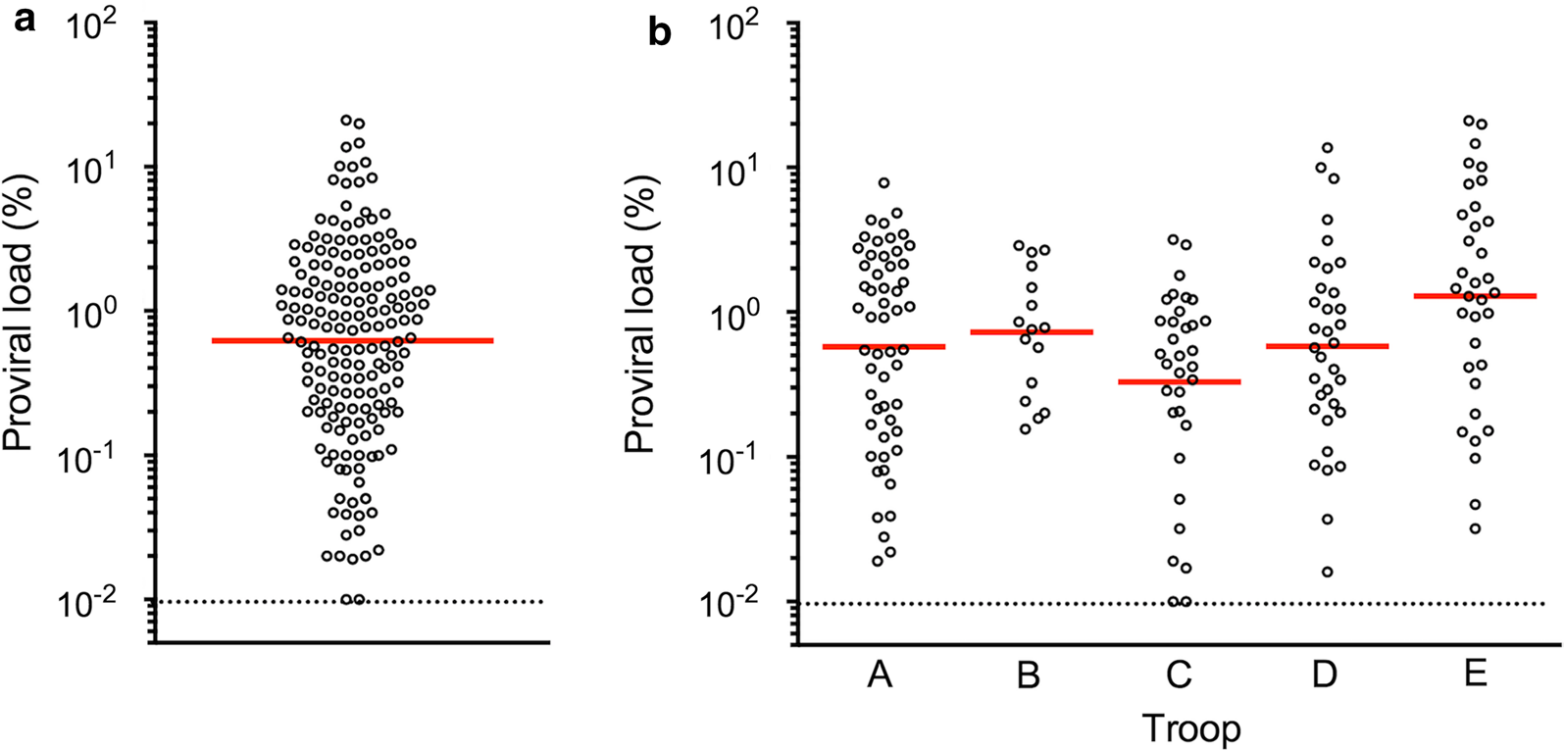
Distribution of proviral loads (PVLs). Distribution of STLV-1 PVLs in all proviral DNA-positive cohorts JMs (**a**) and in each troop (**b**) is shown. The dotted line indicates the detection limit of the PVL (0.01%), and the horizontal line indicates the geometric mean of the PVL distribution

**Fig. 3:**
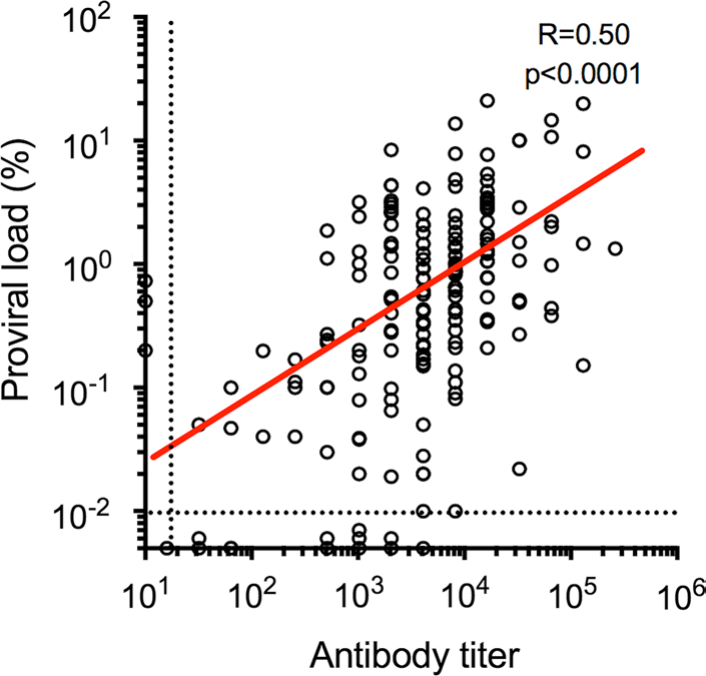
Correlation between antibody titers (ABTs) and proviral loads (PVLs) among individuals who were positive for either value. Among the JMs (N = 183), 168 were positive for both values, whereas three were seronegative but positive for PVLs, and 12 were seropositive but negative for PVLs. The horizontal and vertical dotted lines show the detection limits of PVL and ABT, respectively, as indicated in Figs. 1, 2. There was a significant correlation between the ABTs and the PVLs (R = 0.50; p < 0.0001)

We hypothesized that a substantial proportion of STLV-1-infected JMs might play a critical role as viral super-spreaders for frequent horizontal transmission and eventual high prevalence of STLV-1, possibly because of their abnormally high viral loads and poor humoral immune response against STLV-1. In fact, our recent incidence of an outbreak of infectious malignant thrombocytopenia in JMs by simian retrovirus type 4 (SRV-4) demonstrated that some of the monkeys developed asymptomatic SRV-4 infection with persistent viremia in the absence of SRV-4-specific antibody response and became viral super-spreaders [54, 55]. Taking this unexpected result into account, we evaluated ABTs and PVLs in the JM cohort. We found that the ABTs among 180 seropositive macaques were normally distributed with a geometric mean of 4076 and an ABT of 8192 at the maximum number of individuals (Fig. 1a, Additional file 1: Figure S1). We observed no significant difference in the titers among the five troops (Fig. 1b). We also examined the STLV-1 PVLs in PBMC samples and found that the PVLs of 171 proviral DNA-positive macaques were normally distributed and ranged from 0.01 to 20% with a geometric mean of 0.62% and PVLs from 0.64 to 1.28% at the maximum number of individuals (Fig. 2a, Additional file 2: Figure S2). Again, we observed no significant difference in the PVLs among the troops (Fig. 2b). The data regarding ABTs and PVLs from the 183 macaques positive for either value (herein tentatively regarded as ‘STLV-1-infected’) were plotted as shown in Fig. 3. Among the JMs, 168 were positive for both values, whereas three were negative for ABTs but positive for PVLs and 12 were positive for ABTs but negative for PVLs. Among the STLV-1-infected macaques, we did not observe any individuals with abnormally high PVLs and poor ABTs (Fig. 3). It is notable that the three ABT^−^PVL^+^ monkeys belonged to either of two troops (two macaques in troop C and one in troop D), and their PVLs were comparable or less than the mean PVLs. It is therefore unlikely that only three monkeys caused the high prevalence in all the independent troops. Rather, we observed positive correlation between ABTs and PVLs (R = 0.50, p < 0.0001) (Fig. 3), suggesting that humoral immunity was properly induced in response to the increasing proviral loads in these macaques.

We then sought the possible route(s) of transmission by which this high prevalence occurred. It was previously reported the age-related increase of STLV-1 prevalence in JMs, suggesting the frequent horizontal transmission whereas the mode of transmission remains to be elucidated [41, 43, 44, 52]. If mother-to-child transmission (MTCT) were the main route, the infection rate should drastically increase at around 1 year of age, followed by a gradual increase with age. On the other hand, if horizontal transmission were the main route, the infection rate should be low in younger ages, followed by a steep increase with age. To verify these possibilities, we examined the age-dependent change of seroprevalence in the cohort. The frequencies of seropositive individuals in each age group were 17%, 33%, 58%, 79%, 93%, 100%, and 95% at age groups of 0, 1, 2, 3–5, 6–8, 9–11, and ≥ 12 years, respectively (Table 2). We also analyzed the age-dependent change of proviral DNA prevalence and found that the frequencies of proviral DNA-positive individuals in each age group were 11%, 31%, 58%, 75%, 89%, 98%, and 91% for the respective age groups (Table 2). The infection rate was over 30% at 1 year of age and was dramatically increased over 50% at the age of 2 years, over 70% at the ages of 3–5 years, followed by infection among almost all of them over 9 years of age, irrespective of the either value of positivity. Considering that the rate of MTCT in the case of long-term breastfeeding is approx. 20% when the children of 3 years old and over of HTLV-1 carrier mothers were tested [56], these results appeared that STLV-1 may be frequently transmitted via both maternal and horizontal routes. Of note, large numbers of younger JMs with relatively low prevalence (i.e., among 280 JMs cohort, 102 JMs were 0**–**1 years of age) reduced the apparent prevalence rate of the entire cohort (64%), although almost all the adult JMs elder than 9 years of age were positive (Table 2). In addition, there was no significant difference among troops regarding ABTs and PVLs (data not shown). We also examined the dynamics of the ABTs and PVLs with age among the STLV-1-infected JMs. It was found that the ABTs and PVLs were gradually increased from 0 to 2 years of age, followed by a slight increase of ABTs and mostly stable PVLs with age (Table 2).

**Table 2:** Age-dependent changes of the prevalence of anti-STLV-1 antibody and provirus DNA in JMs

Age	Number of JMs examined	Number of seropositive	Seropositive %	Geometric mean of antibody titer	Number of PVL-positive	Proviral DNA-positive %	Geometric mean of PVL
0	54	9	17	93	6	11	0.046
1	48	16	33	2048	15	31	0.42
2	31	18	58	2702	18	58	0.74
3–5	28	22	79	3846	21	75	0.53
6–8	27	25	93	3769	24	89	0.38
9–11	48	48	100	5087	47	98	0.54
12 ≤	44	42	95	6619	40	91	1.2
Total	280	180	64		171	62	

Results described above suggested that STLV-1 was frequently transmitted via both maternal and horizontal routes. However, it is still possible to speculate that the JMs of long-term (e.g. more than 3 years old) latent MTCT but not horizontal transmission might gradually convert to be positive, which led to the result as shown in Table 2. In order to validate this possibility, we conducted a retrospective study of the STLV-1 seroprevalence in this cohort. First, the cohort during the period from 2011 to 2012 was examined for the seroprevalence and found that 224 of 366 JMs (61%) were seropositive (PBMC samples during this period were not available), which was mostly comparable with the result during the period from 2015 to 2016 (64%) as shown in Table 1. We then selected 139 monkeys whose plasma samples were available in both periods. In 2011–2012, 111 of 139 JMs were seropositive, whereas 28 were seronegative. Four years later, 24 (86%) of the seronegative 28 monkeys in the former period were seroconverted for the antibody (Table 3). Remarkably, among ten seronegative monkeys of 4 years of age and older (i.e., sexually mature monkeys) during the period from 2011 to 2012, eight were seroconverted within 4 years interval (80%), which was comparable with the monkeys of 3 years of age and younger (i.e., sexually immature monkeys) in the former period (16/18, 89%). Although seroconversion occurred mostly in the younger ages, the frequency of seroconversion between the seronegative immature and mature monkeys was comparable (89% and 80%, respectively), which was consistent with the results shown in Table 2. These results strongly suggest frequent STLV-1 transmission occurring among JMs via both horizontal and maternal transmission routes.

**Table 3:** Longitudinal study of the STLV-1 seroprevalence in JMs

Age in 2011 (Age in 2015)	Number of seronegative in 2011	Number of seropositive in 2015	Frequency of seroconversion
0 (4)	7	6	
1 (5)	1	1	
2 (6)	6	6	
3 (7)	4	3	
0–3 (4–7)	18	16	16/18 (89%)
4 (8)	3	3	
5 (9)	1	1	
6 (10)	3	3	
≥ 7 (≥ 11)	3	1	
≥ 4 (≥ 8)	10	8	8/10 (80%)
Total	28	24	24/28 (86%)

It has been shown that a certain degree of heterogeneity of HTLV-1 genome was present among the virus-infected individuals in the same community [57, 58] while the heterogeneity of the viral genome between mother and child was minimal [59–65]. If it is the case with STLV-1-infected JMs, then it could be possible to differentiate whether the STLV-1 infected in a monkey is derived from mother through MTCT or from any other monkeys through horizontal transmission. In order to examine the possibility, we compared the nucleotide sequences of STLV-1 LTR, tax and env regions from randomized 12 JMs in a troop D as a representative. It was found that almost all the sequences of 3′LTR and env region were identical in all the monkeys except for only a unique heterogeneity in some monkeys (for 3′LTR: C333G for A1671; G346A for A2594, for env: T1218C for 8 monkeys, respectively) (Additional file 3: Figure S3a, c). Furthermore, no heterogeneity was observed in the tax region of all monkeys (Additional file 3: Figure S3b). These results indicated that STLV-1 genome was highly conserved among the JMs in the troop, with consistent results in terms of other troops (Kuramitsu et al. unpublished observation). Consequently, it was not possible to determine the transmission route of STLV-1 on the basis of the heterogeneity of the virus genome, which was unlike HTLV-1.

## Discussion

In this study, we aimed to investigate the epidemiological background by which STLV-1 infection is highly prevalent in JMs. We initially examined the prevalence of STLV-1 infection in five independent troops of 280 JMs originating from different areas in Japan. It was found that 64% (180/280) of the macaques were seropositive, which was generally consistent with previous reports [41, 43, 44, 52] (Table 1). Second, the ABTs and PVLs in the STLV-1-infected JMs were normally distributed with mean values of 4076 and 0.62%, respectively, which were mostly comparable to those of HTLV-1-infected humans (Figs. 1, 2). As no macaques exhibiting abnormally high PVLs together with poor ABTs were observed, our initial hypothesis that a small proportion of JMs might be the viral super-spreaders was unlikely (Fig. 3). Third, the frequency of STLV-1 infection was over 50% at the age of 2 years, irrespective of the either value of positivity (Table 2), suggesting the possibility of greater frequency of MTCT as compared with humans. Finally, our retrospective study indicated that frequent seroconversion occurred in not only younger (until 3 years of age, sexually immature) but also older (4 years of age and over, sexually mature) seronegative monkeys (Table 3). Together with the fact that almost all of the full-adult JMs older than 9 years old were infected with STLV-1, our results of this study demonstrated for the first time that frequent horizontal and mother-to-child transmission may contribute to high prevalence of STLV-1 infection in JMs.

Contrary to our speculation, we did not observe the JMs exhibiting aberrant ABTs and PVLs who might contribute to frequent STLV-1 transmission as super-spreaders. It is therefore reasonable to consider that immunological abnormality of a subpopulation of JMs may not be attributed to the high prevalence of STLV-1. It was also possible to assume that the environmental condition in which the JMs in our animal facility might be, at least in part, associated with the high prevalence. In this point of view, the rearing population density was not correlated with the seroprevalence of each troop (Table 1), suggesting that the density did not influence the high prevalence. Moreover, we recently examined the plasma samples of the JMs stored at the quarantine procedure when they were previously introduced in our facility and found that the STLV-1 seroprevalence of them was generally comparable with that of the JMs in the breeding facility as shown in this study (data not shown), indicating the high prevalence of JMs by nature as previously reported [41, 43, 44, 52].

It has been shown that JMs genetically originate in rhesus macaques (RMs) as the ancestor macaques came over from the Asian Continent to Japan around 0.5 million years ago [66] and that much less frequency of RMs are infected with STLV-1 than the case of JMs [67], which is consistent with the fact that the prevalence rate of STLV-1 in RMs bred and reared in our free-range facility is less than 1% [52]. It is therefore reasonable to speculate that STLV-1 was broadly disseminated after ancestor macaques started inhabiting Japan. As for the migrated JMs, foods such as leaves, fruits, and nuts in their habitats were insufficient in the cold winter season so they probably needed to form troops in order to keep their territories for foods and to stay warm by assembling together [68]. Interestingly, RMs are promiscuous in terms of mating [69], as JMs do so without having fixed partners/mates, which may circumvent the genetic disadvantages caused by inbreeding within the troop [70]. It is possible that promiscuity together with the tight troop society might increase the opportunity of sexual transmission, which led to the high STLV-1 prevalence in JMs. In fact, it was reported that a relatively high prevalence of HTLV-1 was occasionally observed in isolated Japanese populations [71] and in Noir-Marron isolated population in French Guiana [72]. Alternatively, severe contact due to fights among the macaques regardless of gender may also be the additional cause of the horizontal transmission.

It was found that the rates of seropositive and proviral DNA-positive JMs were 17% and 11%, respectively (Table 2). The reason that the former was greater than the latter may be the inclusion of the individuals that were still positive for maternal antibody without STLV-1 infection-induced seroconversion. Subsequently, the frequency of STLV-1 infection was over 30% at the age of 1 year and over 50% at the age of 2 years, respectively, as observed in either value (Table 2). These rates were much higher than the case of humans, suggesting that MTCT may occur frequently in JMs. JMs usually breastfeed around 1 year, while some of the mothers feed longer period up to 1.5 years,which is generally comparable with humans. It is therefore reasonable to consider that the length of the infant period may not be the reason for the high MTCT rate. It is also unclear yet whether MTCT is solely responsible for the steep increase in the rate of STLV-1 infection among the younger monkeys until 2 years of ages or not only MTCT but also horizontal transmission due to fighting would contribute to the increase. The longitudinal study of MTCT in a cohort of mother-baby pairs isolated from other monkeys may answer the question.

## Conclusion

Our results of this study demonstrated for the first time that frequent horizontal and mother-to-child transmission may contribute to high prevalence of STLV-1 infection in JMs.

## Supplementary information

**Additional file 1: Figure S1** Distribution of anti-STLV-1 antibody titers (ABTs) in seropositive JMs. The X-axis represents antibody titers ranging from 16–262144, with an ABT of 8192 at the maximum number of individuals. The Y-axis represents the number of individuals in each antibody titer.

**Additional file 2: Figure S2.** Distribution of STLV-1 proviral loads (PVLs) in the JMs positive for STLV-1 proviral DNA. The X-axis indicates PVLs ranging from 0.01%–20%, with PVLs of 0.64%–1.28% at the maximum number of individuals. The Y-axis shows the number of individuals in each PVL group.

**Additional file 3: Figure S3 a, b and c.** Alignments of the STLV-1 partial nucleotide sequences in a JM troop. Total of 12 proviral DNA-positive JMs in troop D were examined as a representative (age ranges: 2-21 years of age; sex: 4 male and 8 female) for the viral sequences of first 600 nucleotides of STLV-1 3’LTR (a) and tax exon3 (b) as well as 1447 nucleotides of entire STLV-1 env (c) by direct sequencing. The results of the alignments demonstrated only one nucleotide variation in both 3’LTR and env but not tax exon3 region for each JMs.

## Data Availability

The datasets used and/or analyzed during the current study are available from the corresponding author on reasonable request.

## Abbreviations

STLV-1: Simian T-cell leukemia virus type 1
HTLV-1: Human T-cell leukemia virus type 1
JM: Japanese macaque
PBMCs: Peripheral blood mononuclear cells
ABT: Antibody titer
PVL: Proviral load
ATL: Adult T-cell leukemia
HAM/TSP: HTLV-1-associated myelopathy/tropical spastic paraparesis
MTCT: Mother-to-child transmission
PA: Particle-agglutination
KUPRI: Kyoto University Primate Research Institute
SRV-4: Simian retrovirus type 4
RM: Rhesus macaque
