# Supplementary figures and images for: Frequent horizontal and mother-to-child transmission may contribute to high prevalence of STLV-1 infection in Japanese macaques
Source: Retrovirology. 2020 Jun 23;17:15. doi: 10.1186/s12977-020-00525-1 (PMC7310504; doi:10.1186/s12977-020-00525-1)

Figure S1

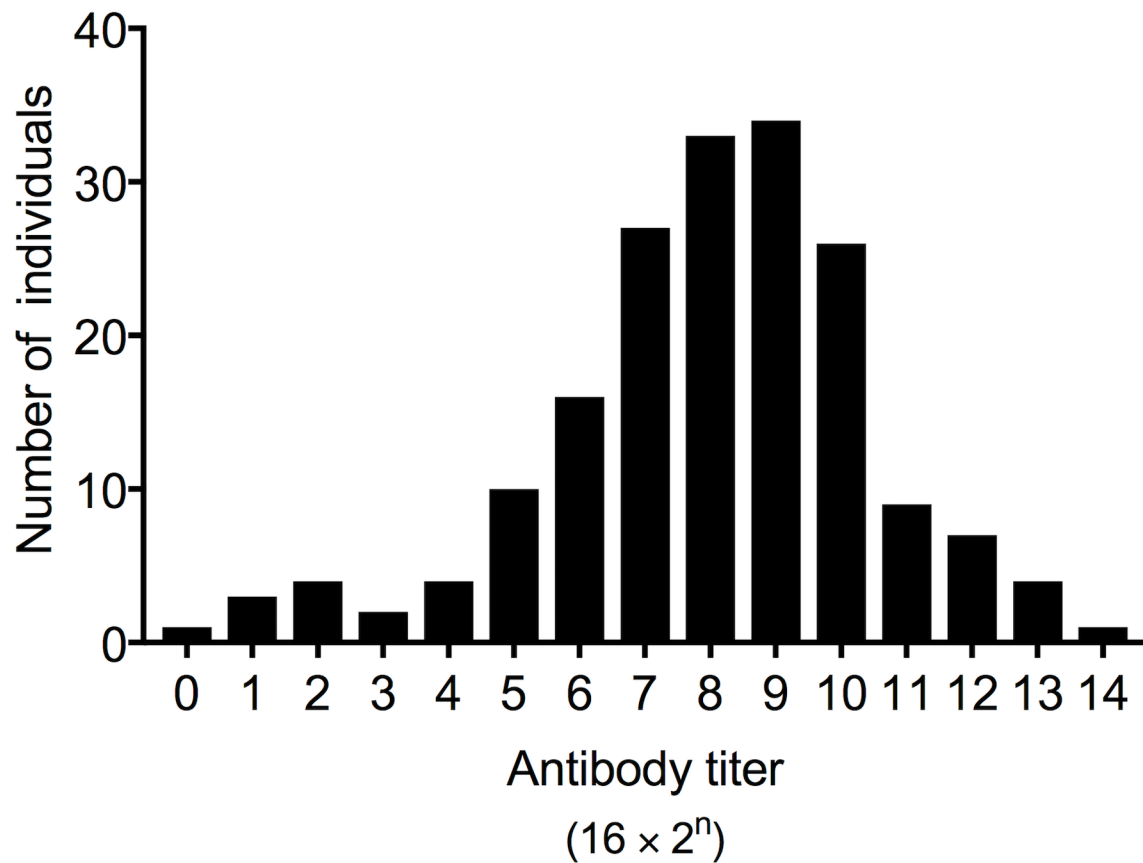

Supplement: Supplementary file 1 — Additional file 1: Figure S1 Distribution of anti-STLV-1 antibody titers (ABTs) in seropositive JMs. The X-axis represents antibody titers ranging from 16–262144, with an ABT of 8192 at the maximum number of individuals. The Y-axis represents the number of individuals in each antibody titer. [file 12977_2020_525_MOESM1_ESM.pdf]
