# Supplementary material for: Frequent horizontal and mother-to-child transmission may contribute to high prevalence of STLV-1 infection in Japanese macaques
Source: Retrovirology. 2020 Jun 23;17:15. doi: 10.1186/s12977-020-00525-1 (PMC7310504; doi:10.1186/s12977-020-00525-1)
Supplement: Supplementary file 2 — Additional file 2: Figure S2. Distribution of STLV-1 proviral loads (PVLs) in the JMs positive for STLV-1 proviral DNA. The X-axis indicates PVLs ranging from 0.01%–20%, with PVLs of 0.64%–1.28% at the maximum number of individuals. The Y-axis shows the number of individuals in each PVL group. [file 12977_2020_525_MOESM2_ESM.pdf]

Figure S2

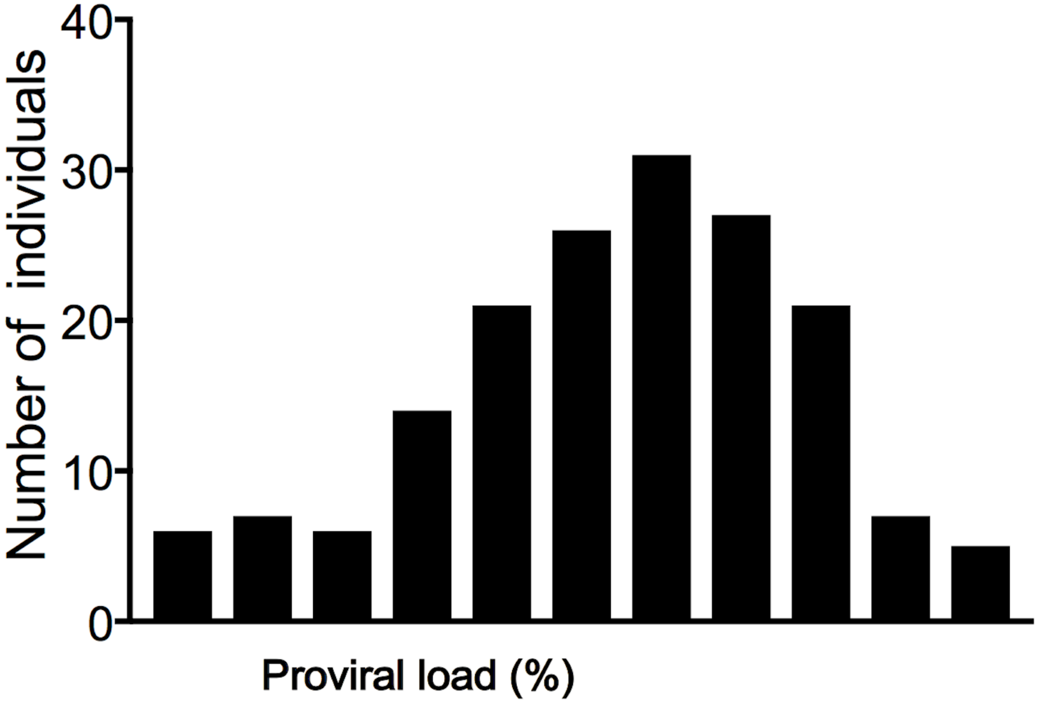

Minimum value of range (more than or equal to)	0.01≤	0.02≤	0.04≤	0.08≤	0.16≤	0.32≤	0.64≤	1.28≤	2.56≤	5.12≤	10.24≤
Value	X	X	X	X	X	X	X	X	X	X	X
Maximum value of range (less than)	<0.02	<0.04	<0.08	<0.16	<0.32	<0.64	<1.28	<2.56	<5.12	<10.24	
