# Supplementary material for: Frequent horizontal and mother-to-child transmission may contribute to high prevalence of STLV-1 infection in Japanese macaques
Source: Retrovirology. 2020 Jun 23;17:15. doi: 10.1186/s12977-020-00525-1 (PMC7310504; doi:10.1186/s12977-020-00525-1)

Figure S3a

```
A1590   1 TGATACTGACCATGAGCCCCAAATGCTCCCCGGGGGCCTAAAGCCTCCCAATGAAAAACATTTCCGCGAAACAGATGTCTGAAAAAGGTCAGGGCCCAGACTAGGGCTCTGACGTCTCCCCCCGGAGGGACAGCTCAGCACCAGGCCCTG 150
A1671   1 ...................................................................................................................................................... 150
A1916   1 ...................................................................................................................................................... 150
A2213   1 ...................................................................................................................................................... 150
A2312   1 ...................................................................................................................................................... 150
A2365   1 ...................................................................................................................................................... 150
A2390   1 ...................................................................................................................................................... 150
A2420   1 ...................................................................................................................................................... 150
A2437   1 ...................................................................................................................................................... 150
A2489   1 ...................................................................................................................................................... 150
A2594   1 ...................................................................................................................................................... 150
A2601   1 ...................................................................................................................................................... 150

A1590 151 ACGTGTCCCCCTGGGGACAAATCATGAACCCAGACCTCCGGGAAGCCACCGGGAACCACCCATTTCCTCCCCATGTTTGTCAAGCCGTCCCCAGGCGTTGACGACAACCCCTCGCCTCAAAAACTTTTTCATGGCACGCATACAGCTCAA 300
A1671 151 ...................................................................................................................................................... 300
A1916 151 ...................................................................................................................................................... 300
A2213 151 ...................................................................................................................................................... 300
A2312 151 ...................................................................................................................................................... 300
A2365 151 ...................................................................................................................................................... 300
A2390 151 ...................................................................................................................................................... 300
A2420 151 ...................................................................................................................................................... 300
A2437 151 ...................................................................................................................................................... 300
A2489 151 ...................................................................................................................................................... 300
A2594 151 ...................................................................................................................................................... 300
A2601 151 ...................................................................................................................................................... 300

A1590 301 TAAAACAACAGGAGTCTATAAAAGCGTGGAGACAGTTCAGGAGGGGGCTAGCTTCTGTTCTACACGCGCCCGCCGCCCTACCTGAGGCCGCCATCCACGCCGGTTGAGTTGCGTTCTGCCGCCTCCCGCCTGTGGTGCCTCCTGAACTGC 450
A1671 301 .............................................A........................................................................................................ 450
A1916 301 ...................................................................................................................................................... 450
A2213 301 ...................................................................................................................................................... 450
A2312 301 ...................................................................................................................................................... 450
A2365 301 ...................................................................................................................................................... 450
A2390 301 ...................................................................................................................................................... 450
A2420 301 ...................................................................................................................................................... 450
A2437 301 ...................................................................................................................................................... 450
A2489 301 ...................................................................................................................................................... 450
A2594 301 ................................G..................................................................................................................... 450
A2601 301 ...................................................................................................................................................... 450

A1590 451 GACCGCCGCCTGGGTAAGTTCGGAGCTCGGGTCGAGACCGGGCCTTTGTCCGGCGCTCCCTTGGAGCCCACCTAGATTCGGCCGGCTCTCCACGCTTTGTCTGACCCTGCTTGCTTAACTCCACATCCTTGTTTCGTTTTCTGTTCCGCG 600
A1671 451 ...................................................................................................................................................... 600
A1916 451 ...................................................................................................................................................... 600
A2213 451 ...................................................................................................................................................... 600
A2312 451 ...................................................................................................................................................... 600
A2365 451 ...................................................................................................................................................... 600
A2390 451 ...................................................................................................................................................... 600
A2420 451 ...................................................................................................................................................... 600
A2437 451 ...................................................................................................................................................... 600
A2489 451 ...................................................................................................................................................... 600
A2594 451 ...................................................................................................................................................... 600
A2601 451 ...................................................................................................................................................... 600
```

Figure S3b

```
A1590   1 CCCATTTCCCAGGTTTTGGACAGAGCCTTCTTTATGGATACCCAGTCTACGTATTTGGAGACTGTGTGCAAGGCGACTGGTGCCCCATCTCTGGGGGACTATGCTCGGCCCGCCTGCACCGTCACGCCCTACTGGCCACCTGTCCAGAGC 150
A1671   1 ...................................................................................................................................................... 150
A1916   1 ...................................................................................................................................................... 150
A2213   1 ...................................................................................................................................................... 150
A2312   1 ...................................................................................................................................................... 150
A2365   1 ...................................................................................................................................................... 150
A2390   1 ...................................................................................................................................................... 150
A2420   1 ...................................................................................................................................................... 150
A2437   1 ...................................................................................................................................................... 150
A2489   1 ...................................................................................................................................................... 150
A2594   1 ...................................................................................................................................................... 150
A2601   1 ...................................................................................................................................................... 150

A1590 151 ATCAGATCACCTGGGACCCCATTGATGGACGCGTTATCGGCTCAGCTCTGCAGTTCCTTATCCCTCGACTCCCCTCCTTCCCCACCCAAAGAACCTCTAAGACCCTCAAGGTCCTTACCCCGCCGGCCACTCATACAACCCCCAACATTC 300
A1671 151 ...................................................................................................................................................... 300
A1916 151 ...................................................................................................................................................... 300
A2213 151 ...................................................................................................................................................... 300
A2312 151 ...................................................................................................................................................... 300
A2365 151 ...................................................................................................................................................... 300
A2390 151 ...................................................................................................................................................... 300
A2420 151 ...................................................................................................................................................... 300
A2437 151 ...................................................................................................................................................... 300
A2489 151 ...................................................................................................................................................... 300
A2594 151 ...................................................................................................................................................... 300
A2601 151 ...................................................................................................................................................... 300

A1590 301 CACCCTCCTTCTTCCAGGCCGTACGAAAATACTCCCCCTTCCGAAACGGATACATGGAGCCCACCCTTGGGCAACAACTCCCAACCCTGTCCTTTCCCGACCCCGGCCTCCGGCCCCAAAACTTGTACACCCTCTGGGGAAACTCCGTTG 450
A1671 301 ...................................................................................................................................................... 450
A1916 301 ...................................................................................................................................................... 450
A2213 301 ...................................................................................................................................................... 450
A2312 301 ...................................................................................................................................................... 450
A2365 301 ...................................................................................................................................................... 450
A2390 301 ...................................................................................................................................................... 450
A2420 301 ...................................................................................................................................................... 450
A2437 301 ...................................................................................................................................................... 450
A2489 301 ...................................................................................................................................................... 450
A2594 301 ...................................................................................................................................................... 450
A2601 301 ...................................................................................................................................................... 450

A1590 451 TCTGCATGTACCTCTACCAGCTTTCCCCCCCCATCACCTGGCCCCTCCTGCCCCATGTAATTTTCTGCCACCCAGGCCAACTTGGGGCCTTTCTCACCAACGTTCCCTACAAACGAATGGAAGAACTCCTCTATAAAATTTTCCTTAATA 600
A1671 451 ...................................................................................................................................................... 600
A1916 451 ...................................................................................................................................................... 600
A2213 451 ...................................................................................................................................................... 600
A2312 451 ...................................................................................................................................................... 600
A2365 451 ...................................................................................................................................................... 600
A2390 451 ...................................................................................................................................................... 600
A2420 451 ...................................................................................................................................................... 600
A2437 451 ...................................................................................................................................................... 600
A2489 451 ...................................................................................................................................................... 600
A2594 451 ...................................................................................................................................................... 600
A2601 451 ...................................................................................................................................................... 600
```

Figure S3c

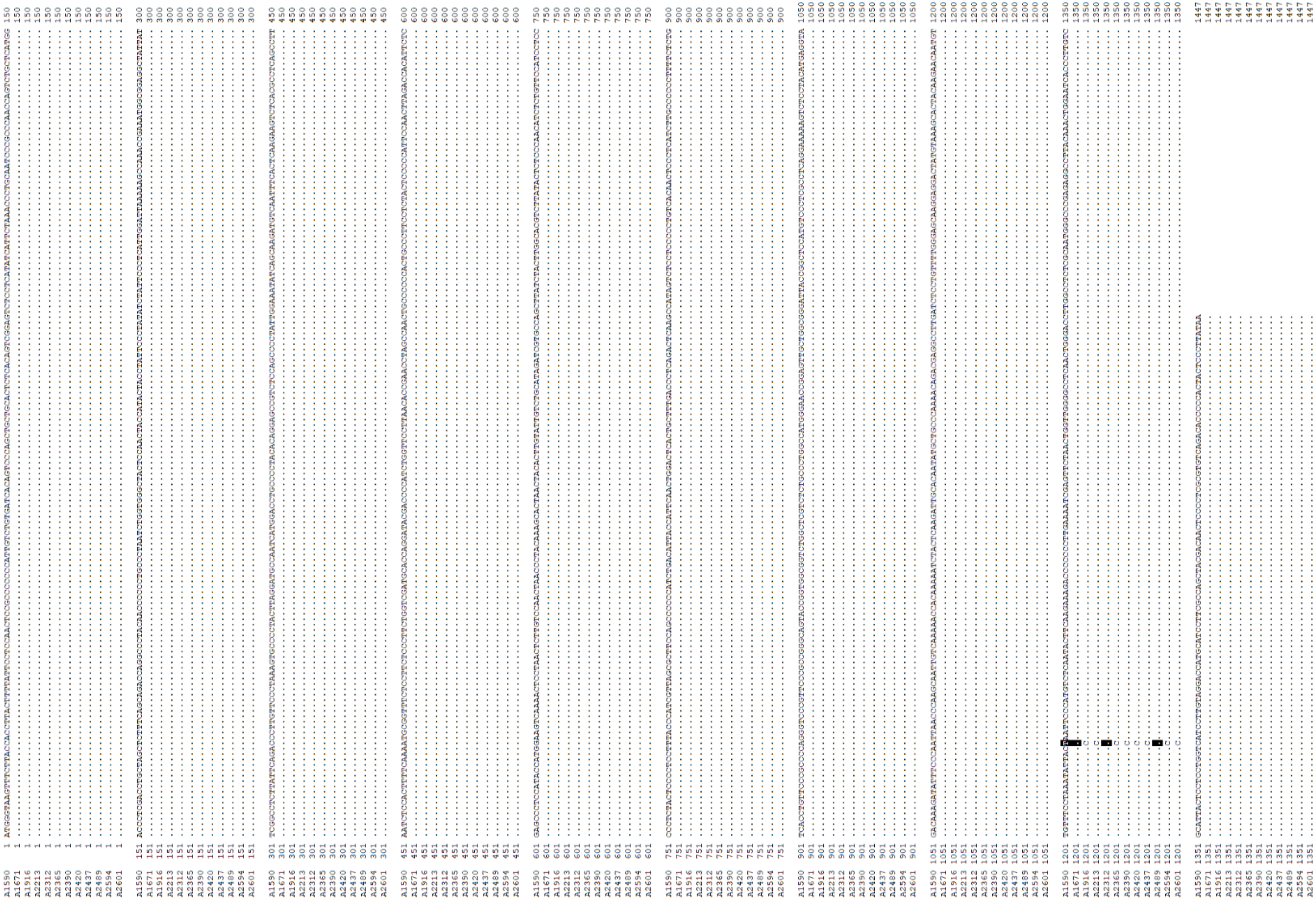

Supplement: Supplementary file 3 — Additional file 3: Figure S3 a, b and c. Alignments of the STLV-1 partial nucleotide sequences in a JM troop. Total of 12 proviral DNA-positive JMs in troop D were examined as a representative (age ranges: 2-21 years of age; sex: 4 male and 8 female) for the viral sequences of first 600 nucleotides of STLV-1 3’LTR (a) and tax exon3 (b) as well as 1447 nucleotides of entire STLV-1 env (c) by direct sequencing. The results of the alignments demonstrated only one nucleotide variation in both 3’LTR and env but not tax exon3 region for each JMs. [file 12977_2020_525_MOESM3_ESM.pdf]
